# Multiple Genetic Lineages and Inter‐Island Gene Flow in *Nemadactylus monodactylus* at Tristan da Cunha: Implications for Phylogeography and Taxonomy

**DOI:** 10.1002/ece3.74452

**Published:** 2026-10-12

**Authors:** Megan Elsmore, Sarah Dalesman, James P. Glass, Jessica Escobar Porras, Mercedes Anne Dorrian‐Smith, Sarah Helyar, Paul W. Shaw, Niall J. McKeown

**Affiliations:** ^1^ Department of Life Sciences Aberystwyth University Aberystwyth UK; ^2^ Tristan da Cunha Fisheries Department Tristan da Cunha Edinburgh of the Seven Seas UK; ^3^ School of Life Sciences University of KwaZulu‐Natal, Westville Campus Durban South Africa; ^4^ School of Biological Sciences Queens University Belfast UK; ^5^ Department of Ichthyology and Fisheries Science Rhodes University Makhanda South Africa

**Keywords:** island biodiversity, phylogeography, population genetics, secondary contact, taxonomy

## Abstract

Tristan da Cunha (TDC) is a remote archipelago of four South Atlantic islands whose nearshore fish assemblages remain comparatively poorly studied. Genetic studies of such disjunct groups can provide valuable information on evolutionary history, taxonomy, and stock structure relevant for conservation and sustainable fishery management. Here, we address such topics in the Five‐finger ( *Nemadactylus monodactylus* ), an abundant nearshore fish harvested for subsistence at TDC, using a combination of mitochondrial control region sequencing and microsatellite analysis. Mitochondrial data, including publicly available data for Southern Hemisphere congeners, recovered four well‐supported clades. Three clades were polyphyletic, containing sequences previously assigned to *N. macropterus* or *N. bergi* , whereas one was newly described and may be endemic to TDC. These patterns add to evidence for a complex evolutionary history within *Nemadactylus* and suggest that current taxonomic designations warrant re‐evaluation. mtDNA also confirms colonisation of TDC by multiple lineages. Neither mtDNA nor microsatellites provided consistent evidence of genetic differentiation among islands, including between the distinctive Gough morphotype and the northern islands. These results are consistent with substantial archipelago‐wide connectivity, but the limited nuclear marker set means that weak structure cannot be excluded. We therefore regard a single archipelago‐wide population as a provisional working hypothesis rather than a confirmed management unit. Broader geographic sampling and genome‐wide nuclear data are needed to test species limits, reconstruct evolutionary history, and resolve fine‐scale population structure relevant to management.

## Graphical abstract

We studied the genetics of the Five‐finger fish across the remote islands of Tristan da Cunha using mitochondrial DNA and microsatellites. mtDNA revealed four genetic clades, three of which were polyphyletic with congeners, and one potentially unique to Tristan da Cunha, suggesting current species classifications may be inaccurate. The data support multiple colonisations of TDC and also that there are high levels of gene flow across the islands supporting a single archipelago‐wide population as a provisional working hypothesis for management.

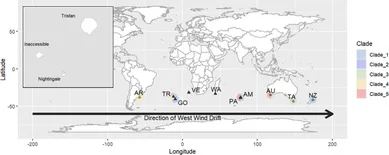

## 1 Introduction

Oceanic islands contribute disproportionately to global biodiversity (DeMartini and Friedlander 2004; Cortés 2012; Friedlander et al. 2013, 2014; Tershy et al. 2015; Kueffer and Kinney 2017; Brodie et al. 2021; Fernández‐Palacios et al. 2021), with isolation and strong selection often promoting endemic species (Whittaker and Fernández‐Palacios 2007; Kier et al. 2009; Tershy et al. 2015; Pinheiro et al. 2017). However, island biotas are often depauperate and characterised by small, isolated populations, making them highly vulnerable to anthropogenic pressures such as biological invasions (Rocha et al. 2015), climate change (Asch et al. 2018), pollution (Perez‐Venegas et al. 2017) and overfishing (Edgar et al. 2010; Friedlander et al. 2013). In this context, inaccurate delineation of biological units can lead to underappreciation of unique regional diversity and can compromise conservation and fisheries management (Ely et al. 2017; Bernatchez et al. 2017). Robust genetic inference can help define management units, evaluate stock sustainability and prevent indiscriminate harvesting (Healey et al. 2020; Silva, Young, et al. 2021).

The Tristan da Cunha archipelago (hereafter TDC) is a remote chain of four volcanic islands (Tristan, Nightingale, Inaccessible, Gough) in the South Atlantic Ocean, over 2000 km from the nearest continental landmass (Figure 1). TDC's nearshore environment provides vital nutritional and economic resources for the local community (Scott 2017). However, research attention has historically focused on high‐profile species (e.g., lobsters, seals, seabirds; Glass 2014; Bester et al. 2019; Steinfurth et al. 2020; Silva, Murphy, et al. 2021; Silva, Young, et al. 2021), and data on less charismatic taxa, particularly nearshore fishes, are lacking. For common reef‐associated fishes of TDC two questions are of central importance. First, do TDC populations represent distinctive evolutionary lineages at the broader biogeographic scale? Second, do intraspecific connectivity patterns among the islands shape a single cohesive stock or is there finer‐scale stock structuring?

**FIGURE 1:**
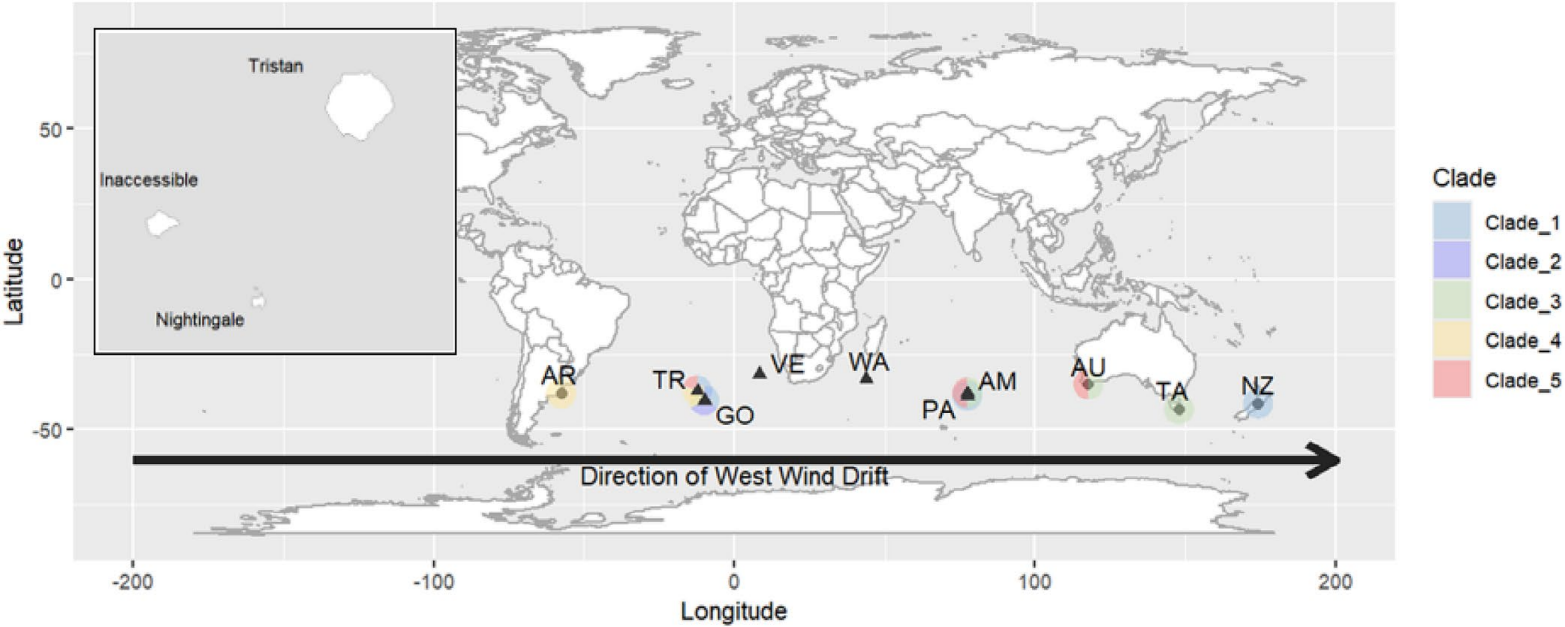
The suggested distribution of southern hemisphere *Nemadactylus*. Black triangles indicate the distribution of *N. monodactylus* as per Scott (2017). Coloured circles represent the location of the five *N. monodactylus* clades (clade 1 = blue, clade 2 = purple, clade 3 = green, clade 4 = yellow, clade 5 (Burridge 1999) = red). Black circles indicate the location of *Nemadactylus* GenBank sequences (Burridge 1999). AM, Amsterdam Island; AR, Argentina; AU, Albany and Australia; GO, Gough; NZ, New Zealand; PA, St. Paul Island; TA, Tasmin Island and Tasmania; TR, Tristan da Cunha top islands (Tristan, Nightingale and Inaccessible); VE, Vema Seamount; WA, Walters Shoal.

The Five‐finger, *Nemadactylus monodactylus* , is one of the most abundant nearshore fish species at TDC (Caselle et al. 2018). The suggested geographic distribution of *N. monodactylus* (Figure 1) spans the South Atlantic (TDC and Vema Seamount), Southwest Indian Ocean (St. Paul and Amsterdam Islands, Walters Shoal) and a single report from the Australasian region (Austral Seamount) (Scott 2017). *N. monodactylus* is one of seven recognised species in the genus *Nemadactylus*, which is estimated to have radiated during the late Pliocene–Pleistocene (~0.6–2.6 Ma; Burridge 1999). The other six *Nemadactylus* species are distributed across the Southern Hemisphere: one endemic to Vema Seamount ( *N. vemae* , Penrith 1967), three Australasian species ( *N. valenciennesi* , Whitley 1937; *N. macropterus* , Forster, 1801 (see Bloch and Schneider 1801); *N. douglasii* , Hector 1875), and two South American species ( *N. gayi* , Kner 1865 in Chile; *N. bergi* , Norman 1937 in Argentina). An additional putative species (‘*Nemadactylus* sp.’ of Smith et al. 1996) has been posited for southern Australian and New Zealand waters (Roberts 2015; Roberts et al. 2020; Papa et al. 2022) but remains formally undescribed. Phylogenetic relationships within *Nemadactylus* are not fully resolved, with a particular dearth of data for *N. monodactylus* at TDC. At TDC, it inhabits rocky reefs to ~150 m depth around all four islands. There are reported morphological and ecological differences between *N. monodactylus* at Gough Island and those at the northern ‘top’ islands (Tristan, Nightingale, Inaccessible; Figure 2). The Gough morphotype has a smaller head and feeds more heavily on invertebrates (consuming algae less frequently), compared to the top‐island fish (Andrew and Hecht 1992). The species is currently caught for subsistence, taken as bycatch, and occasionally used as bait by the islanders (Andrew et al. 1995; Scott 2017; Caselle et al. 2018). However, the importance of *Nemadactylus* fishes as commercial fisheries in other regions (Papa et al. 2022) highlights the potential to expand the fishery of *N. monodactylus* at TDC and reduce the pressure on other groups (Cline et al. 2017). Effective conservation and management of any future *N. monodactylus* fishery requires information as to whether *N. monodactylus* at TDC includes unique evolutionary lineages and whether fish from Tristan, Nightingale, Inaccessible, and Gough comprise one stock or several partially independent management units. Those questions are especially relevant in a remote archipelago where monitoring capacity is limited and where precautionary spatial management may need to operate at the scale of the whole island group.

**FIGURE 2:**
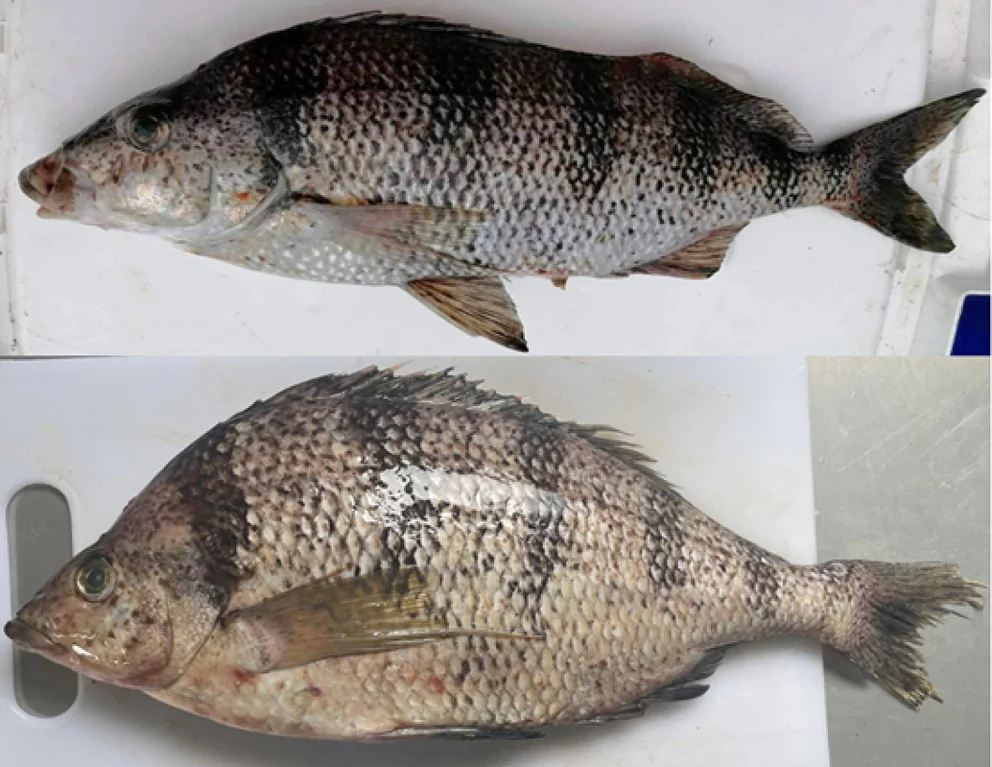
Morphological differences between *N. monodactylus* found at the TDC top islands (Tristan, Nightingale and Inaccessible) (top) and at Gough Island (bottom).

Here, we combine mitochondrial control‐region sequencing and nuclear microsatellite markers to: (i) assess the phylogenetic placement of TDC *N. monodactylus* lineages relative to conspecific populations elsewhere and to congeners ( *N. macropterus* and *N. bergi* ), inferring the likely colonisation dynamics; and (ii) evaluate whether current taxonomic designations are congruent with observed genetic patterns; (iii) test for population genetic structuring within and among the TDC islands (including whether the distinctive Gough morphotype is genetically differentiated). In addressing these objectives, we integrate sequence data and inferences from previous phylogeographic studies of *Nemadactylus* (Burridge 1999; Burridge and Smolenski 2004; Halliwell 2019; Papa et al. 2022). By adding insight into the deep evolutionary history and local connectivity patterns of a dominant reef fish, this study contributes to characterising TDC's biodiversity and a framework for managing such resources under future environmental change.

## 2 Materials and Methods

### 2.1 Sample Collection and DNA Extraction

Fin clip samples of *N. monodactylus* were collected across all four TDC islands during 2021–2022 by the TDC Fisheries Department. Tissue samples were taken from fish caught as bycatch or during subsistence fishing and fisheries surveys, then preserved in 70% ethanol. Total genomic DNA was extracted using a standard cetyltrimethylammonium bromide (CTAB) chloroform/isoamyl alcohol protocol (Winnepenninckx et al. 1993).

### 2.2 Ethics Statement

As the samples were collected by the TDC Fisheries Department, the sampling complied with local regulations and permits.

### 2.3 mtDNA Sequencing and Analysis

A fragment (~379 bp) of the mitochondrial control region (CR) was amplified by Polymerase Chain Reaction (PCR) using primers L15926 (5′‐TCAAAGCTTACACCAGTCTTGTAAACC‐3′; Kocher et al. 1989) and H16498 (5′‐CCTGAAGTAGGAACCAGATG‐3′; Meyer et al. 1990). PCRs were carried out in 10 μL volumes containing ~3 μL DNA template, 5 μL MyTaq DNA Polymerase mix (Bioline, UK), and 0.5 μL of each primer (10 μM). Thermoprofiles consisted of an initial denaturation at 95°C for 3 min; 35 cycles of 95°C for 30 s, 50°C for 60 s, 72°C for 30 s; and a final extension at 72°C for 60 s; followed by a 4°C hold. Amplicons were Sanger‐sequenced (BigDye Terminator chemistry, Applied Biosystems 3730 sequencer) using the L15926 primer.

Raw sequences were edited and trimmed in Chromas v2.6.6 (Technelysium Pty Ltd., 2018) and aligned in BioEdit v7.2.5 (Hall 1999) using CLUSTALW (Thompson et al. 1994). The generated sequences were combined with *Nemadactylus* CR sequences from GenBank ( *N. monodactylus* , plus the closely related species *N. bergi* , and *N. macropterus* ; see Table S1 for accession numbers). Unique haplotypes were identified using DnaSP v6 (Rozas et al. 2017). Phylogenetic relationships among haplotypes were inferred using a median‐joining haplotype network in POPART v1.7 (Bandelt et al. 1999; Leigh and Bryant 2015) and by Maximum Likelihood (ML) tree analysis in MEGA v11 (Tamura et al. 2021). The ML tree used 1000 bootstrap replicates to assess nodal support, with *Nemadactylus douglasii* specified as outgroup (Smith et al. 1996). Model selection in MEGA indicated the Tamura 3‐parameter model with gamma correction (T92 + G) as the best‐fit nucleotide substitution model for these data.

Standard mtDNA genetic diversity indices (haplotype diversity (*h*) and nucleotide diversity (*π*)) were estimated in Arlequin v3.5 (Excoffier et al. 2005). Genetic differentiation between each pair of islands was quantified using pairwise *F*_ST_ with significance assessed via 10,000 permutations in Arlequin. To further evaluate genetic differentiation between Gough and the northern islands, a hierarchical analysis of molecular variance (AMOVA; Excoffier et al. 1992) was performed in Arlequin. The four island samples were assigned to two groups: Gough; and Tristan da Cunha, Inaccessible and Nightingale, with each island retained as a separate population within its respective group. Molecular variance was partitioned into components attributable to differences among groups, among island populations within groups, and within island populations. Statistical significance of variation at each level was assessed using 10,000 permutations.

Past population demographic changes were examined using mismatch distribution analysis: the goodness‐of‐fit of observed pairwise difference distributions to a sudden‐expansion model was evaluated by the sum of squared differences (SSD) and Harpending's raggedness index (HRI; Harpending 1994), with significance determined by 10,000 bootstraps in Arlequin. Expansion times (T in years) were estimated using the formula T = τ/(2 μ) (Rogers and Harpending 1992) where τ is the mismatch distribution's age parameter and μ is the per‐sequence mutation rate. Two CR mutation rates were assessed: a slower rate of 3.6% per Myr (Donaldson and Wilson Jr 1999) and a faster rate of 10% per Myr (Bowen et al. 2006), based on rates used for related species. To further test for deviations from neutral expectations/signatures of historical population size fluctuations Tajima's *D* (Tajima 1989) and Fu's *F*S (Fu 1997) were calculated (for various sample/lineage configurations) with significance evaluated by 10,000 permutations.

### 2.4 Microsatellite Genotyping and Analysis

Seven microsatellite loci previously developed for the related species *N. macropterus* (Burridge 2000; Burridge and Smolenski 2003) were initially tested in *N. monodactylus* (Table S2). Of these, four loci yielded consistent amplification (NMA118, NMA187, NMA245, plus NMA311 which was monomorphic and later excluded). The three polymorphic loci (NMA118, NMA187, NMA245) were used to genotype individuals (total *N* = 160 fish across islands). PCR amplification followed similar conditions as for mtDNA. Genotypes were resolved on an ABI 3730 capillary sequencer and scored using Peak Scanner (Applied Biosystems).

For each locus and island sample, genetic variation was quantified using the number of alleles (*N*_*A*_), allelic richness (*A*_*R*_) standardised for sample size via FSTAT (Goudet 1995), observed heterozygosity (*H*_*O*_), and expected heterozygosity (*H*_*E*_). Departures from Hardy–Weinberg equilibrium (HWE) and for linkage disequilibrium between loci were assessed using exact tests (10,000 dememorization, 10,000 batches, 5000 iterations) in GENEPOP v4.0 (Rousset 2008). Global and pairwise *F*_ST_ values were estimated in GenAlEx v6.5 (Peakall and Smouse 2006). *F*_ST_ values were also estimated with the null allele correction method implemented in FreeNA (Chapuis and Estoup 2007). Variation between Gough and the northern islands was also assessed by AMOVA as for mtDNA. Genetic structure was also analysed using a range of individual based methods. Firstly, the Bayesian clustering method in STRUCTURE v2.3.4 (Pritchard et al. 2000) was used to estimate the most probable number of genetic groups (K) and the assignment of individuals to these groups. Simulations were run for *K* = 1–5 with 10 replicates each under various models: with/without admixture, with/without sampling location as a prior (LocPrior; Hubisz et al. 2009), and with correlated vs. uncorrelated allele frequencies. Each run used 1 × 10^6 Markov Chain Monte Carlo iterations after a burn‐in of 200,000. Models were assessed using L(K) (Pritchard et al. 2000). Second, genetic structuring was also assessed using the discriminant analysis of principal components (DAPC) implemented in ADEGENET (Jombart et al. 2010). Whereas STRUCTURE assigns cluster membership by minimising Hardy–Weinberg and linkage disequilibria within clusters, DAPC employs fewer assumptions and simply maximises differences between groups while minimising differences within groups with the optimal number of groups identified using the Bayesian Information Criterion (BIC). Third, genetic relationships among individuals were explored using factorial correspondence analysis (FCA) of multilocus microsatellite genotypes, implemented in GENETIX version (4.04) (Belkhir et al. 2004).

## 3 Results

### 3.1 Genetic Diversity

After alignment and trimming, the final alignment comprised 82 *N. monodactylus* control‐region sequences of 379 bp length. Within the TDC samples, 46 unique CR haplotypes were identified. Overall haplotype diversity was very high (*h* = 0.96) and nucleotide diversity was also high (*π* = 0.023) when all TDC fish were considered together (Table 1). Haplotype sharing was extensive among the four islands (Figure 3). However, none of the TDC haplotypes were shared with individuals from the comparison sites in the Indian Ocean (St. Paul and Amsterdam Islands). We also found no haplotype sharing between *N. monodactylus* and its congeners ( *N. macropterus* , *N. bergi* ). Phylogenetic analysis revealed that *N. monodactylus* haplotypes at TDC fall into four distinct clades (Figures 3 and 4), three of which also include haplotypes from *N. macropterus* or *N. bergi* (polyphyletic clades), and one clade (clade 2) that is monophyletic for TDC *N. monodactylus* . The largest assemblage, clade 1, contained the majority of new and GenBank deposited TDC sequences, plus *N. monodactylus* from St. Paul Island and one *N. macropterus* from New Zealand (Figures 1, 3, 4). The second‐largest, clade 3, comprised TDC *N. monodactylus* along with additional *N. macropterus* haplotypes from Australia and Tasmania. A fourth group, clade 4, contained TDC *N. monodactylus* haplotypes mixed with *N. bergi* haplotypes from Argentina. Finally, clade 5 (terminology following Burridge 1999) included *N. macropterus* (Australia) and *N. monodactylus* (Amsterdam Island) sequences from GenBank but no TDC sequences; we therefore exclude clade 5 from further analysis as it represents lineages not present at TDC. When considering only TDC‐derived sequences grouped by these phylogenetic clades, haplotype diversity remained high in all clades (range *h* 0.90–1.00; Table 1). Neither the mitochondrial haplotype network nor the ML phylogeny showed island‐specific clustering of *N. monodactylus* haplotypes: instead, haplotypes from Tristan, Nightingale, Inaccessible, and Gough were intermingled in each of the four clades (Figure 3). There was a significant mtDNA *F*_ST_ between Gough and Inaccessible (*F*_ST_ = 0.089, *p* = 0.038; Table 2). However, comparisons of Gough with Tristan or Nightingale yielded no significant mtDNA differentiation (Table 2). Hierarchical AMOVA detected no statistically significant genetic differentiation between Gough and the northern island group comprising Tristan da Cunha, Inaccessible and Nightingale (*F*_CT_ = 0.004, *p* = 0.793).

**TABLE 1:** Genetic diversity, neutrality tests, and mismatch distribution parameters for *N. monodactylus* mitochondrial clades at Tristan da Cunha (CR sequences).

Phylogenetic group	*n*	*h*	*π*	Mismatch SSD (*P*)	*Hri* (*P*)	τ (3.6%)	τ (10%)	Tajima's *D* (*P*)	Fu's *Fs* (*P*)
Clade 1	32	0.9483 ± 0.0135	0.0088 ± 0.0051	0.0007 (0.7395)	0.0207 (0.6760)	113,090	40,712	**−2.0192 (0.0054)**	**−25.2049 (0.0000)**
Clade 2	5	0.9048 ± 0.1033	0.0133 ± 0.0084	0.0411 (0.4096)	0.0816 (0.6814)	155,600	56,016	0.6720 (0.7529)	0.3127 (0.5177)
Clade 3	6	1.0000 ± 0.0962	0.01390 ± 0.0090	0.0299 (0.6165)	0.0978 (0.5368)	194,811	70,132	−0.4583 (0.3799)	−2.0590 (0.0627)
Clade 4	3	1.0000 ± 0.2722	0.0211 ± 0.0169	0.2291 (0.2443)	0.6667 (0.6360)	398,527	143,470	0.0000 (0.7085)	0.9008 (0.4365)
All sequences grouped	46	0.9660 ± 0.0094	0.0238 ± 0.0123	**0.1648 (0.0004)**	0.0116 (1.0000)	50,388	18,140	−1.2441 (0.0837)	**−22.1657 (0.0000)**

*Note:* Mismatch P with *p*‐value for SSD (goodness‐of‐fit to expansion model; *p* < 0.05 indicates deviation); *HRI* = Harpending's raggedness index (*p*‐value in parentheses). τ (years) estimates expansion time for the given mutation rates (CR: 3.6% and 10%/Myr). Tajima's *D* and Fu's *F*S with their *p*‐values are shown (*p* < 0.05 and *p* < 0.02 respectively). Significant values are presented in bold.

Abbreviations: *π*, nucleotide diversity (±SE); *h*, haplotype diversity (±SE); *n*, number of haplotypes.

**FIGURE 3:**
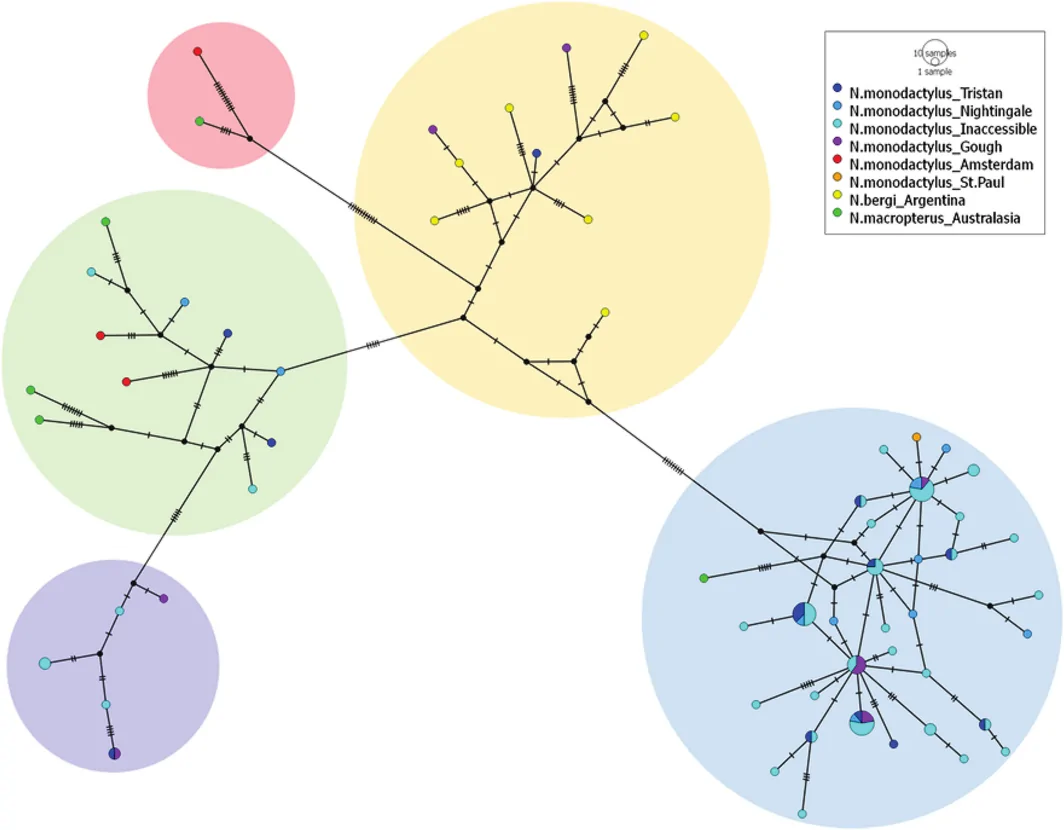
Median‐joining haplotype network for *Nemadactylus* control‐region (CR) sequences. Circle size is proportional to haplotype frequency; colours denote species and location (Tristan top islands, Gough, St. Paul/Amsterdam, Australia, New Zealand, Argentina; see key). Each small dash on connecting lines represents one mutation. Haplotypes are grouped by *N. monodactylus* clade (clades 1–4 circled in blue, purple, green, yellow respectively; clade 5 in red from Burridge 1999). TDC haplotypes occur in clades 1–4 and are shared among islands, indicating little geographic structuring.

**FIGURE 4:**
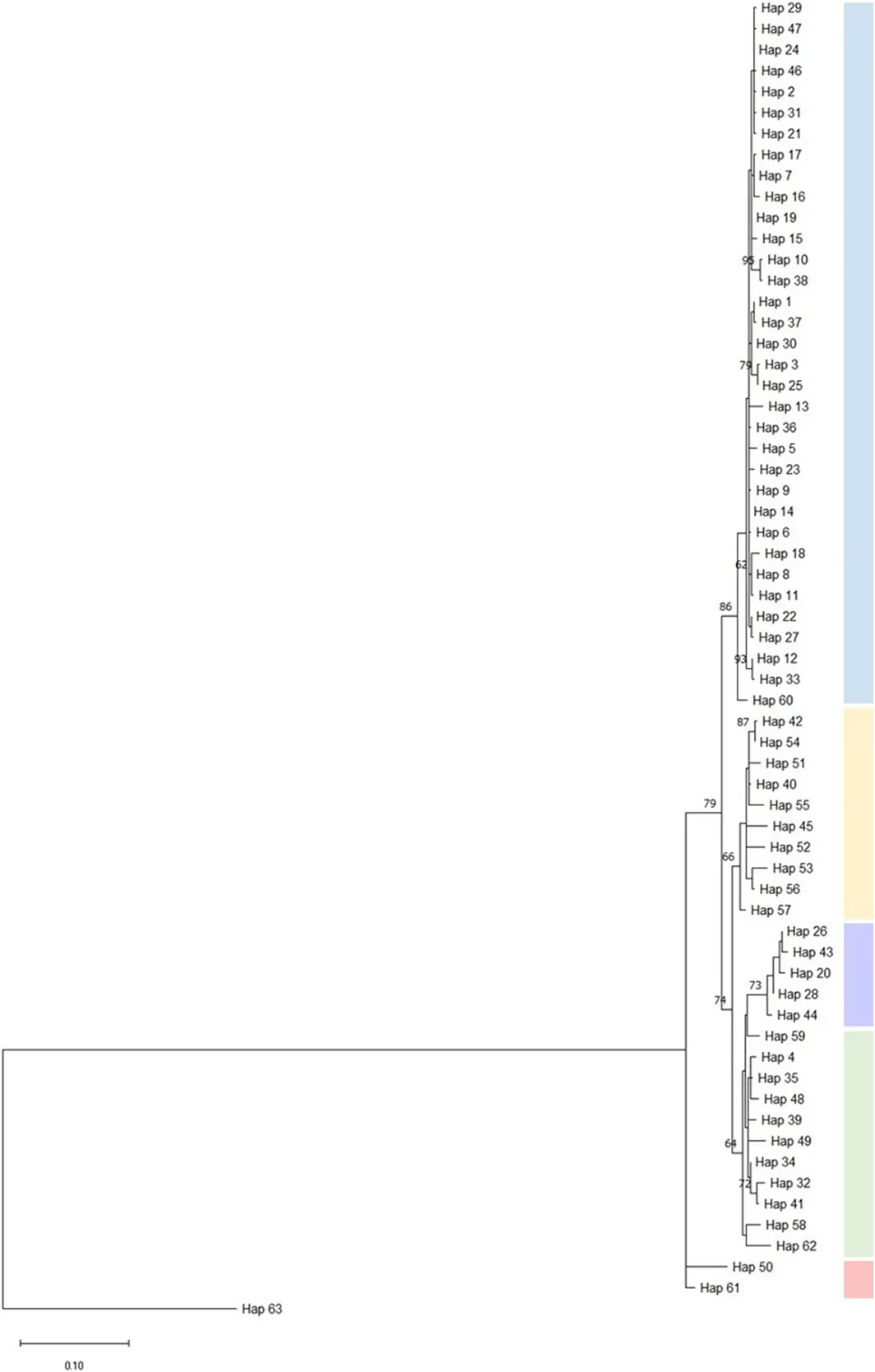
Maximum Likelihood phylogenetic tree of *N. monodactylus* and relatives based on mitochondrial control‐region haplotypes. Bootstrap support > 60% is shown next to nodes. Coloured highlights mark clades 1–5 (clades 1–4 circled in blue, purple, green, yellow respectively; clade 5 in red from Burridge 1999). *Nemadactylus douglasii* (haplotype 63) is the outgroup. The tree illustrates polyphyly of TDC *N. monodactylus* (present in clades 1, 3, 4) and one endemic clade.

**TABLE 2:** Pairwise *F*_ST_ (below diagonal) for *N. monodactylus* among Tristan da Cunha islands based on mtDNA CR haplotypes.

	Nightingale	Inaccessible	Tristan	Gough
Nightingale	—	0.604	0.65	0.274
Inaccessible	−0.019	—	0.203	**0.038**
Tristan	−0.034	0.016	—	0.814
Gough	0.021	**0.089**	−0.048	—

*Note:* *p*‐values for differentiation are above diagonal (significant values in bold). Tristan, Nightingale, Inaccessible (top islands), Gough.

Three microsatellite loci were genotyped across 160 individuals (sample sizes per island: Tristan *n* = 40, Nightingale *n* = 22, Inaccessible *n* = 64, Gough *n* = 34). There was no evidence of linkage disequilibrium between loci (exact tests across all samples; all comparisons *p* > 0.05). The total number of alleles per locus ranged from 2 to 12 (mean alleles per locus = 5.25). Observed heterozygosity ranged from 0.07 to 0.44 depending on locus and island, and all loci showed heterozygote deficits in multiple island samples (Table 3). For each locus, estimated null allele frequencies ranged from 0 to 0.24 (highest in NMA245). Global microsatellite *F*_ST_ was low at 0.004 and statistically non‐significant both with and without ENA correction. Similarly, pairwise *F*_ST_ values between islands were numerically small and statistically non‐significant (Table 4). AMOVA also reported no statistical differences between Gough and the northern island group (*F*_CT_ = 0.012, *p* = 0.425). The STRUCTURE analysis failed to detect genetic substructuring with optimal support for *K* = 1 (*P* ~ 1 and nearly 0 for all other K values tested). Furthermore, at all higher values of *K* examined, individuals did not exhibit preferential assignment to particular clusters (Figure S1). Similarly, BIC supported a single genetic cluster with the DAPC ordination showing extensive overlap among individuals from the different islands (Figure 5). Such overlap was also apparent in the FCA (Figure S2).

**TABLE 3:** Summary of nuclear genetic variation for *N. monodactylus* microsatellite loci at each Tristan da Cunha Island.

Island	Locus	*N*	*N*_a_	*A*_R_	*H*_o_	*H*_e_	*P*_HWE_	*F*
Gough	NMA118	33	4	2.352	0.212	0.196	0.998	−0.082
	NMA187	31	4	2.613	0.129	0.235	0.000***	0.451
	NMA245	8	2	2.000	0.375	0.492	0.501	0.238
Inaccessible	NMA118	52	4	3.262	0.423	0.403	0.236	−0.050
	NMA187	55	7	2.936	0.073	0.265	0.000***	0.725
	NMA245	45	12	5.413	0.444	0.695	0.000***	0.361
Nightingale	NMA118	21	4	2.328	0.182	0.170	1.000	−0.067
	NMA187	19	7	4.376	0.200	0.458	0.000***	0.563
	NMA245	10	5	4.597	0.273	0.682	0.002**	0.600
Tristan	NMA118	41	4	2.689	0.275	0.250	0.985	−0.101
	NMA187	39	7	3.57	0.184	0.366	0.000***	0.497
	NMA245	33	4	3.933	0.364	0.678	0.023*	0.463
Pooled	NMA118	147	4	4.000	0.299	0.285	0.421	−0.049
	NMA187	144	11	9.262	0.132	0.318	0.000***	0.585
	NMA245	75	12	12.00	0.400	0.685	0.000***	0.416

*Note:* Data shown for each locus (NMA118, NMA187, NMA245) per island and overall pooled.

Abbreviations: *AR*, allelic richness (sample‐size corrected); F*, fixation index (inbreeding coefficient); *HO* and *HE*, observed and expected heterozygosity respectively; *N*, sample size; *NA*, number of alleles; *PHWE*, *HWE* exact test *p* (bold indicates significant deviation, *p < 0.05; **p < 0.01; ***p < 0.001).

**TABLE 4:** Pairwise *F*_ST_ results for *N. monodactylus* based on three microsatellite loci at each of the four TDC islands both with (above diagonal) and without (below diagonal) ENA correction.

	Nightingale	Inaccessible	Tristan	Gough
Nightingale	—	0.012	−0.009	−0.002
Inaccessible	0.015	—	0.002	0.004
Tristan	0.014	0.014	—	−0.001
Gough	0.020	0.018	0.021	—

*Note:* All *p*‐values were non‐significant. ENA corrected *p*‐values could not be calculated for the number of loci used.

**FIGURE 5:**
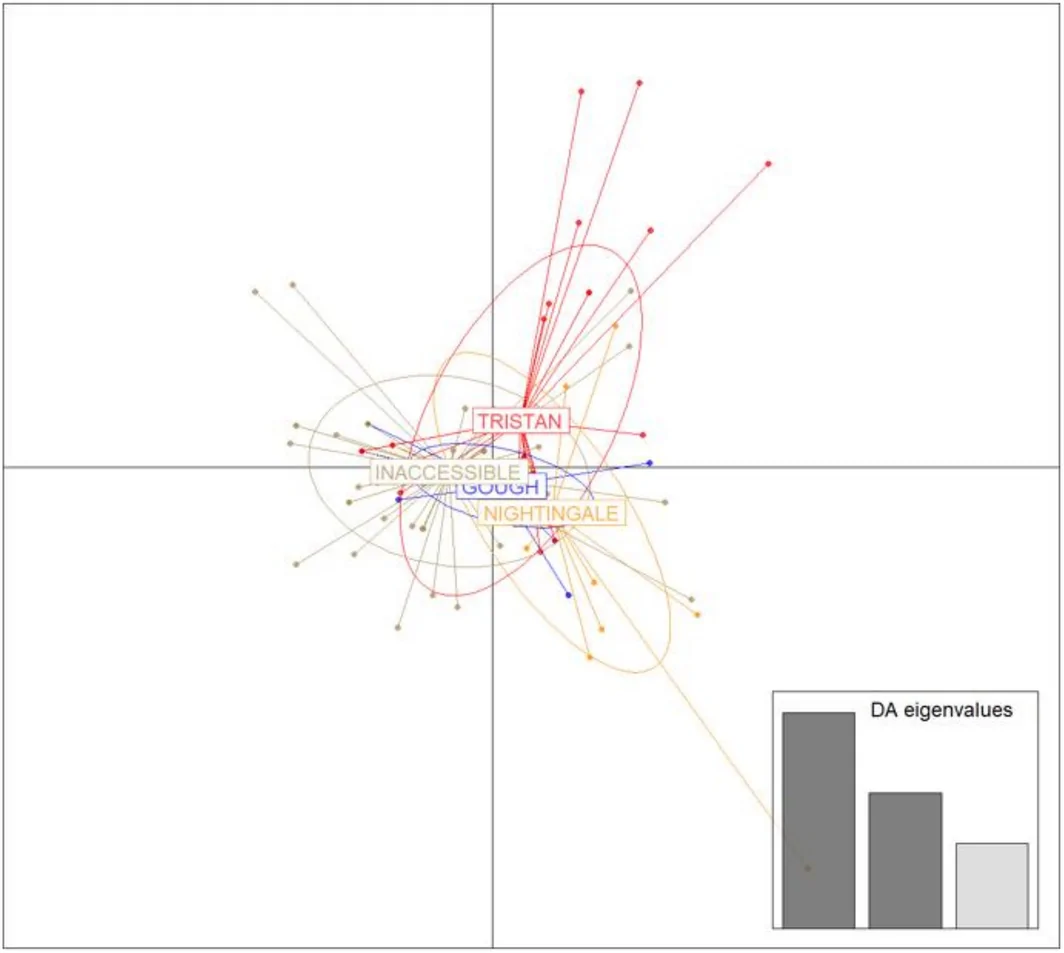
Discriminant analysis of principal components (DAPC) based on multi‐locus microsatellite data from Tristan, Inaccessible, Nightingale and Gough.

### 3.2 Demographic History

MtDNA analyses provided evidence of past population size changes. Mismatch distributions for each phylogenetic clade (clades 1–4) were broadly unimodal and confirmed to the sudden‐expansion model (all SSD *p* > 0.24, HRI *p* > 0.24; Table 1; Figure S3). Neutrality tests were consistent with expansion in several cases (Table 1). Using a CR mutation rate of 3.6%/Myr, the estimated time since expansion (τ) was on the order of ~113,000–398,000 years before present (ybp) for the individual clades (clade 4 expansion being the oldest, Clade 1 expansion the most recent), and ~50,000 ybp when pooling all TDC haplotypes (Table 1). Applying the faster 10%/Myr rate, the expansion timeframes move to ~41,000–143,000 ybp for various clades (Table 1). Clade 1, the most widespread lineage, reported the most recent expansion (~40,000–113,000 ybp under the two rates), whereas clade 4 (with *N. bergi* affinities) expanded earlier (~143,000–398,000 ybp). These expansion estimates all fall within the Pleistocene epoch, and applying a heuristic time‐dependent rate correction (as suggested by Ho et al. 2011) would place the expansions even more recently, overlapping with the Last Glacial Maximum (~18–25 kya) for most clades.

## 4 Discussion

### 4.1 Demographic History: Multiple Origins and Asymmetric Gene Flow

Phylogeographic analyses resolved four divergent mitochondrial clades of *N. monodactylus* at TDC. Three of these (clades 1, 3, 4) showed polyphyletic relationships with continental congeners; specifically, TDC haplotypes were interspersed among *N. macropterus* from Australasia and/or *N. bergi* from South America. The remaining group, clade 2, comprised an endemic Tristan haplotype assemblage not previously reported. The presence of multiple deeply divergent haplotype lineages in the archipelago suggests multiple colonisation events or sources for *N. monodactylus* at TDC (Emerson 2002; Burridge et al. 2006). *N. monodactylus* , *N. bergi* and *N. macropterus* are estimated to have diverged ~0.6–2.6 million years ago (Burridge 1999) during the Pleistocene. The Pleistocene was characterised by cyclical glacial–interglacial oscillations that periodically lowered sea levels, altered current intensities, and changed the permeability of barriers to dispersal (Lambeck et al. 2002; Andrew et al. 1995; Reid et al. 2016) and likely facilitated multiple divergence and colonisation episodes. During glacial maxima, intensified West Wind Drift (WWD) and shifts in the Antarctic Circumpolar Current and Subtropical Convergence could have promoted eastward, asymmetrical larval dispersal from continental source populations ( *N. macropterus* in Australasia, *N. bergi* in South America) to TDC. A west‐to‐east colonisation scenario is consistent with the observed affinities and with previous work on cirrhitoid dispersal (Burridge et al. 2006). The presence of multiple non‐monophyletic lineages at TDC, yet with no shared haplotypes among the three species, aligns with Burridge's (1999) inference of incomplete lineage sorting following recent radiations. In essence, TDC appears to have acted as a ‘melting pot’ where historically separate lineages have arrived and co‐exist.

The data add to evidence suggesting an asymmetric, predominantly eastward, colonisation process. *Nemadactylus* dispersal tends to follow prevailing currents from the west (continental areas) to east (oceanic islands) (Burridge et al. 2006; Cook and Crisp 2005). The polyphyly of TDC haplotypes with Australasian *N. macropterus* (clades 1 and 3) aligns with a southwest Pacific origin of the genus. Under this scenario, *N. macropterus* (in Australasia) would be ancestral to both *N. monodactylus* and *N. bergi* (Burridge 1999). Clade 1, the largest group at TDC, likely represents the first wave to colonise Tristan, possibly diverging from Australasian *N. macropterus* that dispersed via the WWD. That clade 2, which is seemingly endemic to TDC, is closely related to clade 3 suggests it may represent an offshoot of the *N. macropterus* lineage that diverged at Tristan. Alternatively, it might be the remnant of an extinct lineage once present on the continents. Clade 4, linking TDC *N. monodactylus* with Argentine *N. bergi* , provides further evidence of an Austral origin with *N. bergi* likely colonising Tristan later, again via eastward dispersal on the WWD. Notably, we did not observe any mixed *N. bergi* / *N. macropterus* clades. This suggests that these clades only converge at Tristan. The absence of *N. macropterus* haplotypes in Argentina (within *N. bergi* samples) suggests two possibilities: (i) *N. macropterus* never established in South America and bypassed it on the way to TDC, or (ii) it did colonise South America but later died out. As *Nemadactylus* larvae have a pelagic duration of ~12 months and glacial‐period currents were stronger, a direct Australia‐to‐Tristan dispersal is feasible (Burridge 1999). Such a route (without incursion in South America) would explain why *N. bergi* and *N. macropterus* show no historical gene flow and suggest that TDC currently serves as a ‘museum’ preserving *N. macropterus* haplotypes outside their original range.

During Pleistocene glacial periods, lowered sea levels would have exposed seamounts between continents and TDC, creating stepping‐stones for dispersal (Andrew et al. 1995; Pinheiro et al. 2017). As glaciers retreated in interglacial periods, rising sea levels and weakened currents could isolate populations, potentially driving speciation or local extinction (Burridge 1999). Thus, TDC may have alternately served as a cradle (site of new lineage formation), museum (refuge preserving lineages through adverse periods), and grave (site of lineage extinction) of *Nemadactylus* diversity (Rangel et al. 2018; Fernández‐Palacios et al. 2021). Indeed, the repeated recovery of multiple lineages at Tristan is consistent with a dynamic history of colonisation and extinction. For instance, clade 1's presence on Tristan and St. Paul but not in South America today suggests Tristan might have been a ‘grave’ for that lineage's dispersal past the archipelago, with extirpation beyond TDC. Similarly, *N. bergi* haplotypes (clade 4) were not found at St. Paul/Amsterdam, suggesting that TDC marks the end of its eastward spread (a ‘grave’ scenario), or that *N. bergi* did extend to those Indian Ocean islands but subsequently went extinct there. Conversely, the endemic clade 2 could signify Tristan as a ‘cradle’ that generated a unique lineage.

The demographic analyses support population expansions in the late Quaternary for *N. monodactylus* at TDC. Using standard mitochondrial mutation rates, these expansions likely occurred during mid‐to‐late Pleistocene glacial cycles (roughly 50–400 k ybp). If we account for time‐dependent rate overestimation, the timing could shift closer to the Last Glacial Maximum (~20 k ybp). In either case, Pleistocene climate fluctuations had major impacts on *N. monodactylus* effective population sizes. During glacial maxima, TDC likely remained unglaciated (Preece et al. 1986; Griffiths and Waller 2016) and may have served as a refuge while continental habitats contracted. The archipelago's extensive kelp forests in colder periods (Graham et al. 2003) could have provided rich foraging grounds for *Nemadactylus*, buffering them against some climatic stresses.

### 4.2 Conservation and Management Considerations

*N. monodactylus* at TDC appears to reflect multiple historical colonisations from Southern Hemisphere source regions, followed by demographic expansion and secondary contact. Despite environmental differences among islands and the marked separation of Gough from the northern islands, we found no convincing evidence of within‐archipelago genetic subdivision with the current marker set. Neither mtDNA nor the three polymorphic microsatellite loci provided consistent evidence of differentiation among islands. This pattern is consistent with substantial connectivity and with previous indications of movement or connectivity from distribution and mark‐recapture observations (Andrew et al. 1995). The lack of strong subdivision is notable given Gough Island's isolation and its cooler environmental regime. Many Tristan nearshore fishes do not occur at both Gough and the northern islands, suggesting that the oceanographic front and associated gradients can limit dispersal (Andrew et al. 1995; Scott 2017). The lack of detected differentiation here requires cautious interpretation. Firstly, power to detect weak differentiation depends strongly on marker number and informativeness, sample size, and the magnitude of true differentiation (Ryman et al. 2006), and genome‐wide data have resolved weak differentiation in *N. macropterus* that earlier microsatellite analyses did not detect (Papa et al. 2022). Moreover, genetic homogeneity does not necessarily imply demographic interchangeability, because dispersal sufficient to homogenise neutral variation may still be insufficient to replenish locally depleted populations on fisheries‐management timescales (Palsbøll et al. 2007; Lowe and Allendorf 2010; McKeown, Arkhipkin, and Shaw 2017). An archipelago‐wide population is therefore a reasonable provisional working hypothesis, but confirmation of management‐scale connectivity will require higher‐resolution genomic markers, temporal replication, and complementary movement or demographic data (Cadrin et al. 2023; Chemshirova et al. 2023; McKeown, Hauser, and Shaw 2017). Broader genome‐wide and geographic sampling will also be important for reconstructing the species' evolutionary history and testing taxonomic hypotheses. From a conservation perspective, a precautionary priority is therefore to maintain abundance throughout the archipelago and avoid local depletion at individual islands while preserving the divergent mitochondrial diversity present at TDC. Any future development of a *N. monodactylus* fishery would also need to operate within the existing Tristan da Cunha fisheries and marine‐protection framework, alongside precautionary monitoring of island‐level abundance.

## Author Contributions

**Megan Elsmore:** conceptualization (equal), data curation (equal), formal analysis (equal), investigation (lead), methodology (lead), project administration (lead), writing – original draft (equal), writing – review and editing (equal). **Sarah Dalesman:** supervision (equal), writing – review and editing (equal). **James P. Glass:** conceptualization (equal), investigation (equal), resources (equal), supervision (equal). **Jessica Escobar Porras:** investigation (equal), methodology (equal). **Mercedes Anne Dorrian‐Smith:** writing – review and editing (equal). **Sarah Helyar:** writing – review and editing (equal). **Paul W. Shaw:** supervision (equal). **Niall J. McKeown:** conceptualization (equal), formal analysis (equal), funding acquisition (equal), project administration (equal), resources (equal), supervision (equal), writing – original draft (equal), writing – review and editing (equal).

## Funding

This work was supported by Aberystwyth University, AberDoc Scholarship. Fisheries Society of the British Isles, FSBI‐RG21‐206.

## Conflicts of Interest

The authors declare no conflicts of interest.

## Supporting information


**Data S1:** Sequence alignment.

**Figure S1:** STRUCTURE barplots showing the assignment patterns for individuals under models for *K* = 2, 3 and 4.
**Figure S2:** FCA plot showing the relationship among individuals colour‐coded according to island Gough–Yellow; Inaccessible–Blue; Nightingale–White; Tristan–Grey.
**Figure S3:** Mismatched distribution graphs for the four *N. monodactylus* phylogenetic groupings at TDC. Clockwise from top left: clade 1, clade 2, clade 3 and clade 4.
**Table S1:** Accession numbers for GenBank control region sequences from closely related *Nemadactylus* species. *Outgroup.
**Table S2:** Microsatellite loci tested (from Burridge 2000) for *N. monodactylus*. Working loci are indicated in bold.

## Data Availability

All the required data are uploaded as Supporting Information and will also be available on publicly accessible repositories.
