# Supplementary material for: Multiple Genetic Lineages and Inter‐Island Gene Flow in Nemadactylus monodactylus at Tristan da Cunha: Implications for Phylogeography and Taxonomy
Source: Ecol Evol. 2026 Oct 12;16(10):e74452. doi: 10.1002/ece3.74452 (PMC13657860; doi:10.1002/ece3.74452)
Supplement: Supplementary file 1 [file ECE3-16-e74452-s001.docx]

Figure S1: STRUCTURE barplots showing the assignment patterns for individuals under models for K=2, 3, and 4.

Figure S2: FCA plot showing the relationship among individuals colour-coded according to island (Gough – Yellow; Inaccessible - Blue; Nightingale – White; Tristan – Grey.

Supplementary materials

**Figure S3:** Mismatched distribution graphs for the four N. monodactylus phylogenetic groupings at TDC. Clockwise from top left: clade 1, clade 2, clade 3, clade 4.

**Table S1:** Accession numbers for GenBank control region sequences from closely related *Nemadactylus* species. *Outgroup.

| GenBank accession number | Species | Location |
| --- | --- | --- |
| AF067117 | *Acantholatris (Nemadactylus) monodactylus* | Gough Island |
| AF067116 | *Acantholatris (Nemadactylus) monodactylus* | Tristan Island |
| AF067115 | *Acantholatris (Nemadactylus) monodactylus* | Saint Paul Island |
| AF067114-112 | *Acantholatris (Nemadactylus) monodactylus* | Amsterdam Island |
| AF067102-108 | *Nemadactylus bergi* | Argentina |
| AF072876 - AF072877,  AF067100 | *Nemadactylus macropterus* | Australia |
| AF067101 | *Nemadactylus macropterus* | New Zealand |
| AF067099 | *Nemadactylus macropterus* | Tazmania |
| AF067098 | **Nemadactylus douglasii* | Australia |

**Table S2:** Microsatellite loci tested (from Burridge 2000) for N. monodactylus. Working loci are indicated in bold.

| Locus name | Repeat motif | GenBank | Primer Sequences |
| --- | --- | --- | --- |
| Nma106 | (AC)_16_ | AF125115 | GCACTTCATGT ACATGCAGGGTTTT  TAAGCGGCATCTTGAGTGTCTGG |
| **Nma118** | (TCA)_9_ | AF125116 | CAAAAAGCAGCTCTACAGTGACAG CAGAGACAGTTTAGGGAAGTGAAGAC |
| **Nma187** | (AC)_13_(AG)_4_ | AF125117 | GCAACTTCCCCGAGCATCATTA AGAGCCTGCAATT AGAGTCAACCAA |
| Nma230 | (GT)_15_ | AF125118 | AGTTTCCCCCTGCCTACA CCTGAACCACTGCGACACTG |
| **Nma245** | (GT)_21_ | AF125117 | TTCTTAAAGGGCGAGTGATGCTA ATGAAAGATGAAGTGATGGAAACAGAC |
| Nma305 | (GT)_7_AT(GT)_9_ | AF125119 | GATCAGGCTCTTCCAGTTGTCATTCC GTGTCGGCGTTCAGAGGCATCC |
| Nma311 | (AT)_8_ | AF125120 | ACTCCGTCTGTACTCTTTGTTGA CTCAGGCTGCAGGTGGTC |
